# Role of the Oxethyl Unit in the Structure of Vegetable Oil-Based Plasticizer for PVC: An Efficient Strategy to Enhance Compatibility and Plasticization

**DOI:** 10.3390/polym11050779

**Published:** 2019-05-01

**Authors:** Jihuai Tan, Bowen Liu, Qinghe Fu, Liwei Wang, Junna Xin, Xinbao Zhu

**Affiliations:** 1 College of Chemical Engineering, Nanjing Forestry University, Nanjing 210037, China; jihuai.tan@wsu.edu (J.T.); Bowen_Liu1228@163.com (B.L.); fuqinghe@njfu.edu.cn (Q.F.); 2 School of Mechanical and Materials Engineering, Composite Materials and Engineering Center, Washington State University, Pullman, WA 99164, USA; liwei.wang@wsu.edu (L.W.); junna.xin@wsu.edu (J.X.)

**Keywords:** bio-based plasticizer, PVC, polyethylene glycol monomethyl ether, dimer acid, vegetable oil

## Abstract

Developing vegetable oil-derived primary plasticizers for poly(vinyl chloride) (PVC) is still a challenge because of their insufficient compatibility. As described in this work, we report the synthesis of plasticizers through the esterification of polyethylene glycol methyl ether and dimer acid, in which dimer acid is renewable material prepared via a two-step reaction (1) the hydrolysis of fatty acids from soybean oil at 70 °C and (2) subsequent Diels–Alder reaction at 250 °C. The resulting plasticizers, dimer acid-derived polyethylene glycol methyl ether esters (DA-2n, 2n = 2, 4, 6 or 8 referring to the number of oxethyl units per molecule), were blended with PVC. It was found that the tensile properties, transparency, and thermal stability of plasticized PVC (PVC-DA-2n) increased significantly with an increase in the number of oxyethyl units. Fourier-transform infrared spectroscopy analysis revealed that its good compatibility can be attributed to the strong interaction between oxyethyl units and PVC. As the number of the oxyethyl units of plasticizer increased, the glass transition temperature (*T*_g_) of the corresponding plasticized PVC samples decreased from 62.3 (PVC-DA-2) to 35.4 °C (PVC-DA-8). Owing to the excellent plasticization of DA-8, the performances of PVC-DA-8 were comparable or better than that of the PVC plasticized using commercial dioctyl terephthalate (DOTP). The simple but efficient method of this study provides a new avenue for the preparation of vegetable oil-based plasticizers for PVC.

## 1. Introduction

Poly(vinyl chloride) (PVC) has been widely used in various products due to its excellent durability, high versatility, and contamination resistance [1]. However, unmodified PVC resin is rigid, brittle, and shows high melt viscosity. Thus, it must be properly plasticized to meet the requirements of processing and applications. To date, there are more than 100 types of plasticizers in the market with a production of approximately 6.4 million tons/year. Most of them are used for the PVC industry [2]. In particular, phthalates including dioctyl phthalate (DOP) and dibutyl phthalate (DBP) comprise more than 80% of all PVC plasticizers because of its balanced performance and moderate price [3]. However, these low molecular weight phthalates are toxic and are susceptible to migrating to the surface of the products, which leads to the potential harmful effects to humans and the environment [4,5]. Recently, dioctyl terephthalate (DOTP) which has a lower toxicity, has been considered as a potential replacement for phthalate. Still, it is non-renewable and difficult to degrade biologically. In this context, it becomes very urgent to develop green PVC plasticizers that are bio-based, of low toxicity, and biodegradable.

Vegetable oils are potential feedstocks for bio-based plasticizers due to their abundance in nature, low toxicity, biodegradability, and characteristic structures [6,7,8,9,10,11,12]. Furthermore, the double bonds of vegetable oil can be conveniently functionalized to generate multifunctional plasticizers. For example, through epoxidation of the double bond in cardanol derivatives [7,8], dimer acid methyl esters [9], or rice bran oil [10], the resulting products can not only act as plasticizers but also have thermal stabilizing effects for PVC. By introducing a phosphate group into soybean oil [11] or castor oil [12], the resulting plasticizer was rendered with a certain flame retardancy. However, except for a few cardanol derivatives, most of them can only be used as secondary plasticizers due to their poor compatibility with PVC.

One way to increase the compatibility between vegetable oil-based plasticizers and PVC is to increase the polarity of the plasticizer by introducing various polar groups into its structure. Ang et al. [13] developed a novel palm oil-based plasticizer (shown in Scheme SIa), a polycarboxylate, using the alcoholysis of palm oil with glycerol, followed by esterification with phthalic anhydride. The resulting product can be used as a primary plasticizer for PVC. After sequentially undergoing addition, oxidation, and esterification, the new oleic acid esters-based plasticizers containing sulfonyl and carboxylate polar groups (shown in Scheme SIb) could be independently used to replace DOP [14]. However, the processes for the synthesis of these vegetable oil-based products are tedious, and the synthesized intermediate products are difficult to isolate from the reaction system. This limited their commercialization.

In a recent study, we found that the number of ether bonds can efficiently affect the compatibility between petro-based plasticizers and the PVC matrix [15]. Also, a lot of literature has proven that polyether can enhance the self-plasticization of PVC [16,17,18]. We envisioned that, via direct esterification of readily available dimer acid with a polyethylene glycol monomethyl ether, the resulting bio-based plasticizers would also have adjustable polarity, compatibility, and plasticization. Thus, in this work, we designed and synthesized a series of novel dimer acid-derived polyethylene glycol methyl ether esters with different oxyethyl units. This was done with a one-step method using commercial dimer acid and polyethylene glycol ether as feedstocks. After being blended with PVC, the effects of the number of oxyethyl units in dimer acid esters on the properties of PVC samples were investigated. To the best of our knowledge, there has been no study focusing on the synthesis and use of dimer acid-derived polyethylene glycol methyl ether esters in PVC plasticization. This study provides a new and simple method for the preparation of primary plasticizers from vegetable oil feedstocks.

## 2. Methods and Materials

### 2.1. Materials

Poly(vinyl chloride) (DG-1000K) was obtained from the Tianjin Dagu Chemical Co., Ltd., China. Soybean oil was supplied by Anqing Hongyu Chemical Co., Ltd., Anqing, China. Ethylene glycol monomethyl ether, diethylene glycol monomethyl ether, triethylene glycol monomethyl ether, and tetraethylene glycol monomethyl ether were supplied by Jiangsu Yida Chemical Co., Ltd., Jiangyin, China. Other reagents such as montmorillonite catalyst, lithium carbonate, phosphoric acid, cyclohexane (≥99.5%), *p*-toluenesulfonic acid (≥98.0%), DOTP (98.0%), zinc stearate (CP grade), and calcium stearate (CP grade) were purchased from Sinopharm Chemical Reagent Co., Ltd., Shanghai, China. All reagents were used as received.

### 2.2. Synthesis of Dimer Acid and Dimer Acid-Derived Polyethylene Glycol Methyl Ether Esters

#### 2.2.1. Preparation of Dimer Acid (DA)

In this work, DA was chosen as the model compound, and it was synthesized according to the previous reports [19,20]. First, the fatty acids were obtained via the hydrolysis of soybean oil. Typically, 16.0 g of sodium hydroxide and 140 mL of ethanol-H_2_O solution (1:1, *V*/*V*) were added into 1-L three-necked round-bottomed flask with a magnetic stirrer and heated to 70 °C. Then 100.0 g of soybean oil was dropwise added into the above solution with a dropping funnel under the 350 r/min at 70 °C. After a 2 h reaction, the pH of the reaction solution was adjusted to 2~3 using 1 mol/L aqueous hydrochloric acid (HCl) solution. The reaction continued for another 1 h at 70 °C. After cooling down to room temperature, the organic phase was extracted with ethyl ether and then washed with deionized water (DI water) three times. Finally, ethyl ether and water were removed by vacuum distillation to yield 86.7 g yellow liquid. Gas chromatography–mass spectrometry (GC–MS) results indicated that the mixture of fatty acids contained C16:0 (18.1%), C16:1 (0.1%), C18:0 (2.3%), C18:1 (25.8%), C18:2 (50.1%), C18:3 (1.7%), and residues (1.9%). Next, 35.0 g of the mixed fatty acids, 3.0 g of montmorillonite (8.57 wt % on fatty acids) as a catalyst, 0.175 g of lithium carbonate (0.5 wt % on fatty acids) and 0.032 g H_3_PO_4_ (0.09 wt % on fatty acids) as co-catalysts, and 0.7 g of distilled water (2 wt % on fatty acids) were added sequentially into a high-pressure autoclave. Prior to a reaction, the system was purged using pure N_2_ gas to remove air. The reaction was performed out at 250 °C for 8 h while undergoing stirring. When the temperature was decreased to room temperature, the catalyst and unreacted fatty acids were removed by filtration and vacuum distillation, respectively. Finally, 21.3 g of yellowish viscous product was obtained. High-performance liquid chromatography (HPLC) results indicated that the product was made up of 85.1 wt % of dimer acid and 14.9 wt % of trimer acid. The acidity and viscosity of the product at 25 °C were 198.8 mg/g and 6900 mPa.s, respectively. The number average molecular weight (Mn) of the product was 651 g/mol and its molecular weight distribution (i.e., polydispersity index, Mw/Mn) was 1.5. The ^1^H NMR (400 MHz, CDCl_3_,) results were 2.5 (m, 1H), 2.4 (t, *J* = 6.9 Hz, 4H), 1.9 (s, 2H), 1.7–1.5 (m, 6H), 1.3 (d, *J* = 17.5 Hz, 33H), and 0.9 (m, 6H); the ^13^C NMR (100 MHz, CDCl_3_) results were 180.5, 34.3, 32.1, 29.8–29.2, 24.8, 22.9, and 14.3.

#### 2.2.2. Synthesis of Dimer Acid-Derived Polyethylene Glycol Methyl Ether Esters (DA-2n)

**Scheme 1:**
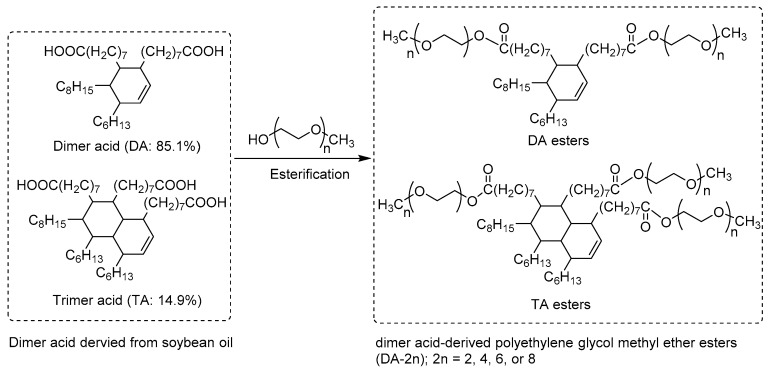
Schematic synthetic route of dimer acid-derived polyethylene glycol methyl ether esters (DA-2n).

DA-2n were synthesized by direct esterification between dimer acid and the corresponding polyethylene glycol methyl ether (Scheme 1). Typically, 112.2 g of dimer acid (0.2 mol), polyethylene glycol methyl ether (0.56 mol), 1.1 g of *p*-toluenesulfonic acid as catalyst (1.0 wt % based on dimer acid) and 20.2 g of cyclohexane as an azeotropic dehydrating agent (18.0 wt % based on dimer acid) were charged into a four-neck round bottom flask equipped with a magnetic stirrer, a temperature-controller, and a Dean–Stark trap with a condenser. The reaction was conducted at 140 ~ 150 °C for 5 h while undergoing stirring. After the reaction, cyclohexane and the excess polyethylene glycol methyl ether were removed via distillation under a vacuum. The remaining liquid was neutralized to a pH of 7 with a saturated NaHCO_3_ aqueous solution, decolorized with active carbon at 90 °C for 1 h, filtrated, and finally dried by distillation under a vacuum.

Dimer acid di(methoxy ethanol)ester (DA-2). The acidity and viscosity of DA-2 at 25 °C were 0.05 mg KOH/g and 80 mPa.s, respectively. The ^1^H NMR (400 MHz, CDCl_3_) results were 4.2 (t, 4H), 3.6 (m, 4H), 3.4 (s, 6H), 2.5 (m, 1H), 2.3 (t, *J* = 7.6 Hz, 4H), 2.0 (s, 2H), 1.9 (s, 1H), 1.6 (s, 7H), 1.3 (d, *J* = 11.4 Hz 38H), and 0.8 (m, 7H); The ^13^C NMR (100 MHz, CDCl_3_) results were 174.2, 63.6, 59.3, 34.5, 32.2, 30.0, 25.3, 23.03, and 14.5.

Dimer acid di(2-(2-methyloxyethoxy)ethanol)ester (DA-4). The acidity and viscosity of the DA-4 at 25 °C were 0.09 mg KOH/g and 140 mPa.s, respectively. The ^1^H NMR (400MHz, CDCl_3_) results were 4.2 (m, 4H), 3.7 (m, 4H), 3.6 (m, 4H), 3.5 (m, 4H), 3.4 (s, 6H), 2.5 (m, 1H), 2.3 (t, *J* = 7.6 Hz, 4H), 2.0 (s, 1H), 1.6 (m, 6H), 1.3 (d, *J* = 10.5 Hz, 38H), and 0.9 (m, 6H); The ^13^C NMR (400MHz, CDCl_3_) results were 173.8, 71.9, 70.4, 69.2, 63.3, 59.0, 34.2, 31.9, 29.3-29.1, 24.9, 22.7, and 14.1.

Dimer acid di(2-(2-(2-methoxyethoxy)ethoxy)ethanol)ester (DA-6). The acidity and viscosity of DA-6 at 25 °C were 0.08 mg KOH/g and 150 mPa.s, respectively. The ^1^H NMR (400MHz, CDCl_3_) results were 4.2 (m, 4H), 3.7 (m, 5H), 3.6 (m, 12H), 3.5 (m, 4H), 3.4 (s, 6H), 2.5 (m, 1H), 2.3 (t, *J* = 7.6 Hz, 4H), 2.0 (s, 2H), 1.7-1.5 (m, 7H), 1.3 (d, *J* = 10.5 Hz, 38H), and 0.9 (m, 6H). The ^13^C NMR (100MHz, CDCl_3_) results were 173.8, 71.9, 70.6, 70.5, 69.2, 63.3, 59.0, 34.2, 31.9, 29.7-29.1, 24.9, 22.7, and 14.1.

Dimer acid di(2-(2-(2-(2-methoxyethoxy)ethoxy)ethoxy)ethanol)ester (DA-8). The acidity and viscosity of DA-8 at 25 °C were 0.04 mg KOH/g and 190 mPa.s, respectively. The ^1^H NMR (400 MHz, CDCl_3_) results were 4.2 (m, 4H), 3.7-3.6 (m, 24H), 3.6 (m, 4H), 3.4 (s, 6H), 2.5 (m, 1H), 2.3 (t, *J* = 7.6 Hz, 4H), 2.0 (s, 3H), 1.8 (s, 1H), 1.7-1.6 (m, 5H), 1.3 (d, *J* = 10.4 Hz, 38H), and 0.9 (m, 6H); The ^13^C NMR (100MHz, CDCl_3_) results were 173.8, 71.9, 70.5, 69.2, 63.3, 59.0, 34.2, 31.9, 29.6-29.1, 24.9, 22.6, and 14.1.

### 2.3. Preparation of Plasticized PVC Test Samples

Test specimens were prepared using a three-step process. Firstly, PVC powder (100 g), plasticizer (50 g) and thermal stabilizers (2.0 g mixed Ca soap and Zn soap with a 3:1 ratio) were pre-mixed using a mechanical mixer at room temperature for 30 min. Secondly, the mixture was compounded at 170 °C for 5 min using a two-roll mill (XK, Changzhou Dongfanghuayang Machinery Company, Changzhou, China). Finally, the test bars were produced according to different standards by the through pressing in the curing press (XLB-D350, Shanghai Rubber Machinery Co., Ltd., Shanghai, China) at 175 °C for 15 min.

### 2.4. Characterizations

Nuclear magnetic resonance (NMR) spectra were recorded on a Bruker spectrometer at a frequency of 400 MHz (Bruker, Germany) using CDCl_3_ as a solvent. The Fourier-transform infrared (FTIR) absorption spectra (4000–400 cm^−1^) of the samples were recorded using a Thermo Nicolet Nexus 670 spectrometer (Nicolet, Thermo Scientific) with a resolution of 4.0 cm^−1^ for 64 scans per sample. The composition of fatty acids was determined by GC–MS (TRACE DSQ, Thermo) equipped with a DB-5MS capillary GC column (30 m (length) × 0.25 mm (inner diameter) × 0.25 μm (film thickness)). HPLC (Waters HPLC, Model Alliance e2695) was used for the analysis of the content of the dimer acid following the method described in the literature [21].

The molecular weight of dimer acid was obtained by using a Viscotek gel permeation chromatography (GPC) system (Malvern Instruments, Malvern, UK) equipped with a GPCmax™ pulp and a TDA 305 multi-detector. The columns used for GPC separation were two AM GPC gel columns (500 Å, 5 µm) in series. Tetrahydrofuran (THF) was used as an eluent at a flow rate of 1.0 mL/min.

The transmittance of the plasticized PVC was determined using a UV-vis spectrophotometer (Perkin−Elmer, Model Lambda 25, with a wavelength of 500 nm, Waltham, MA, USA). The fracture surfaces of plasticized PVC samples were examined using VEGA3 LMU SEM (Tescan Ltd., Brno, Czech Republic).

The tensile test was performed in accordance with ASTM standard D638, type V. All tests were carried out on a SANS CMT-4303 universal testing machine (Shenzhen Xinsansi Jiliang Instrument Co., Shenzhen, China). The cross-head speed was set at 10 mm/min. Five specimens per blend were tested to obtain an average value. All test bars were conditioned in a desiccator for a minimum of 96 h prior to tensile testing.

Thermogravimetric analysis was performed on a TGA/DSC1/1100SF instrument (Mettler Instruments Ltd., Zurich, Switzerland) in N_2_ atmosphere (50 mL/min). Each sample was scanned from 30 to 600 °C at a heating rate of 10 °C/min. Dynamic mechanical properties of PVC samples were measured using a dynamic mechanical analyzer (DMA) Q800 machine (TA Instruments, New Castle, DE, USA) at a frequency of 1 Hz in a single-cantilever mode. Specimens for the DMA test were rectangular with the dimensions 35 mm (L) × 13 mm (W) × 3 mm (T). The scanning temperature was set from −40 to 120 °C at a rate of 3 °C/min.

Volatility was determined according to the ASTM D2199-82 standard. The PVC samples (20 × 20 × 1 mm^3^) were placed in a convection oven (Shanghai Suopu Instrument Co., Shanghai, China) at 70 °C for 24 h, then cooled to room temperature in a desiccator for 2 h. The weight changes were measured before and after heating, according to Equation (1):

(1) $$\mathrm{Weight}\;\mathrm{loss}=\frac{W_{1}-W_{2}}{W_{1}}\times 100$$

A leaching test was carried out according to the ASTM D1239 standard. Specimens (20 × 20 × 1 mm^3^) were respectively immersed in distilled water, hexane, 30% (*w*/*w*) NaOH aqueous solution, 30% (*w*/*w*) acetic acid aqueous solution, 50% (*w*/*w*) ethanol aqueous solution and epoxidized soybean oil at 23 ± 2 °C and 50 ± 10% relative humidity for 24 h. After immersion, those specimens were rinsed and dried in an oven at 50 °C for 24 h. The weight loss, before and after immersion, was calculated according to the Equation (1).

The biodegradation experiments involving DA-8 and DOTP were performed following previous reports [22,23,24]. The microorganism *Rhodococcus rhodocrous* (BeNa Culture Collection, Beijing, China) was selected as the bacteria to simulate the biodegradation of plasticizer in the environment, and the detailed degradation process was described elsewhere [24]. Briefly, 100 mL of minimum mineral salt medium containing 6 g/L Na_2_HPO_4_, 4 g/L NH_4_NO_3_, 4 g/L KH_2_PO_4_, 0.2 g/L MgSO_4_.7H_2_O, 0.014 g/L Na_2_EDTA, 0.01 g/L CaCl_2_. 2H_2_O, 0.01 g/L FeSO_4_.7H_2_O, 10 mmol/L pure plasticizer, 2 g/L hexadecaneand, and 0.1 g/L yeast extract were combined in a 500 mL Erlenmeyer flask equipped with a foam cap. The above solution was autoclaved at 115 °C and 100 kPa for 30 min and cooled. Then 1 mL of cell broth was inoculated into the flask via sterile techniques. After that, the flask was cultured in an incubator-shaker at 30 °C and 120 rpm. After biodegradation for fourteen days, the solution was disposed of using H_2_SO_4_ and the resulting products were extracted by chloroform. The structure of each compound and its concentration in the mixture was determined using a gas chromatographer equipped with a flame ionization detector (GC-9800, Shanghai Kechuang Chromatography Co., Ltd., Shanghai, China) and GC–MS, respectively.

## 3. Results and Discussion

### 3.1. Synthesis and Characterizations of DA and DA-2n

**Figure 1:**
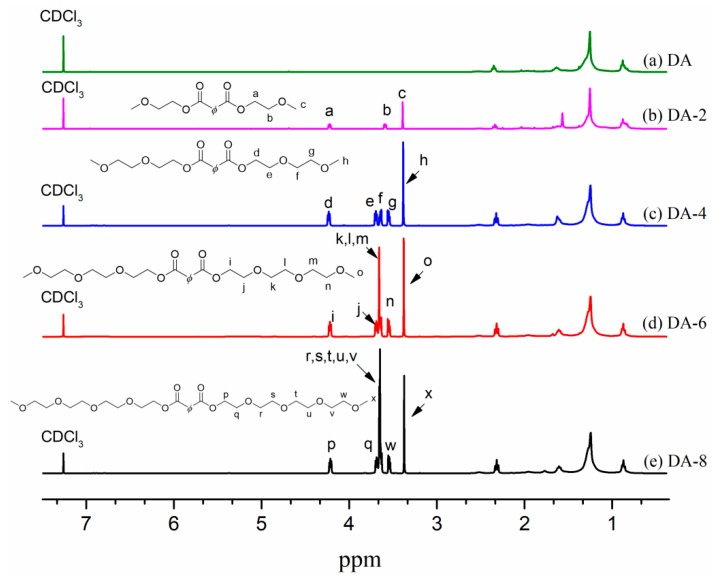
^1^H NMR spectra of (**a**) DA, (**b**) DA-2, (**c**) DA-4, (**d**) DA-6, and (**e**) DA-8. Note: *ø* represents residues of DA, except for carboxylate.

As shown in Scheme SII, dimer acid synthesized from soybean oil is a mixture, containing multiple diastereoisomers of dimer and trimer acids. Due to all esters of those diastereoisomers could be used as plasticizers, the separation of those acids is unnecessary in this study. The mixed acid was still labeled as DA. As shown in Figure 1a, Figure S1a, and Figure S2a, the relative clean NMR spectra of DA were obtained, which might be attributed to the similar chemical shifts of those diastereoisomers. Similar results have been reported by Zheng [25] and Jia [26], where waste cooking oil was directly used as feedstock to prepare a new bio-based plasticizer. 

The formation of dimer acid-derived plasticizer (DA-2n, Scheme 1) could be evidenced from the changes in NMR spectra, where cyclic dimer and trimer acids were chosen as representatives of DA to react with polyethylene glycol methyl ether. Figure 1b–e showed the appearance of new peaks at 4.2 ppm and 3.5–3.75 ppm were detected. These peaks could be assigned to methylene protons connected with the carboxyl group and methylene protons from the oxyethyl units, respectively (Figure 1b–e), indicating the successful formation of ester products.

**Figure 2:**
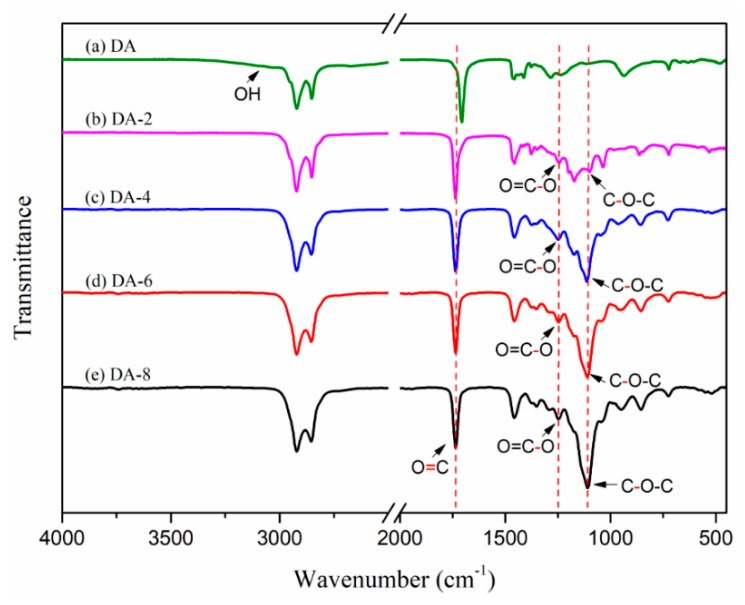
FT-IR spectra of DA and DA-2n.

To further confirm the esterification, FTIR technique was carried out to characterize the structures of pristine DA and DA-2n (Figure 2). It was found that the board band for the hydroxyl groups of DA at 3080 cm^−1^ disappeared, while the typical peaks of carboxylic ester groups at 1736 cm^−1^ (O=C) and 1248 cm^−1^ (O=C–O–C) appeared. Also, new peaks at 1100–1130 cm^−1^ attributed to the absorption of ether bonds (C–O–C) were observed in DA-2n. This evidence indicates the successful esterification of the dimer acid and polyethylene glycol methyl ether.

### 3.2. FTIR Characterization of the PVC Samples Plasticized with DA-2n

**Figure 3:**
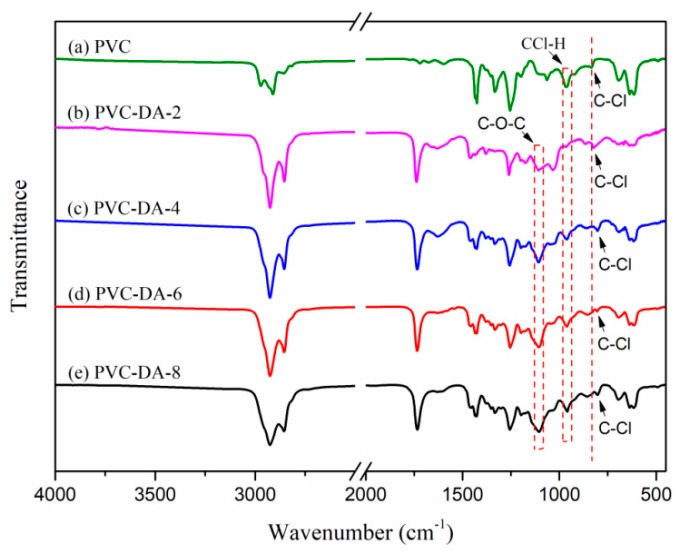
FT-IR spectra of (**a**) PVC, (**b**) PVC-DA-2, (**c**) PVC-DA-4, (**d**) PVC-DA-6, and (**e**) PVC-DA-8.

The interaction between dimer acid-derived polyethylene glycol methyl ether esters (DA-2n) and PVC matrix was determined by FTIR. In the FTIR spectrum of neat PVC (Figure 3a), all the characteristic peaks of PVC; the peaks of the asymmetrical stretching vibration at 2974 cm^−1^; the symmetrical stretching vibration of C–H of CHCl at 2910 cm^−1^; the bending vibration of CH_2_ aliphatic at 1433 cm^−1^, CCl–H out-of-plane angular deformation at 1257 cm^−1^, C–C stretching at 1065 cm^−1^; C–H out-of-plane transverse deformation at 964 cm^−1^; and C–Cl bond stretching at 834, 694, and 615 cm^−1^ were consistent with what was reported in the literature [27]. For the DA-2n plasticized PVC samples (with a plasticizer/PVC weight ratio of 50:100), the bands of CCl–H at 964 cm^−1^ and C–Cl at 833 cm^−1^ were both shifted to low wavenumbers with the increase in the number of oxyethyl units. Figure S3a provides more details on this. Hooke’s law shows that the force constant is proportional to the wavenumber. When oxyethyl units were introduced into the structure of plasticizer, it efficiently formed hydrogen bonds with the PVC skeleton, resulting in the weakening of the intermolecular bonding of PVC. Chen et al. [28] also observed that the wavenumber of CH–Cl shifted to a low position when the polar epoxy group from cardanol-based plasticizer interacted with the PVC matrix. The shift of the absorption peak from 1108 to 1105 cm^−1^ (detail is shown in Figure S3b) associated with the ether groups in DA-2n also proves the existence of hydrogen bonding.

### 3.3. Dynamic Mechanical Properties

**Figure 4:**
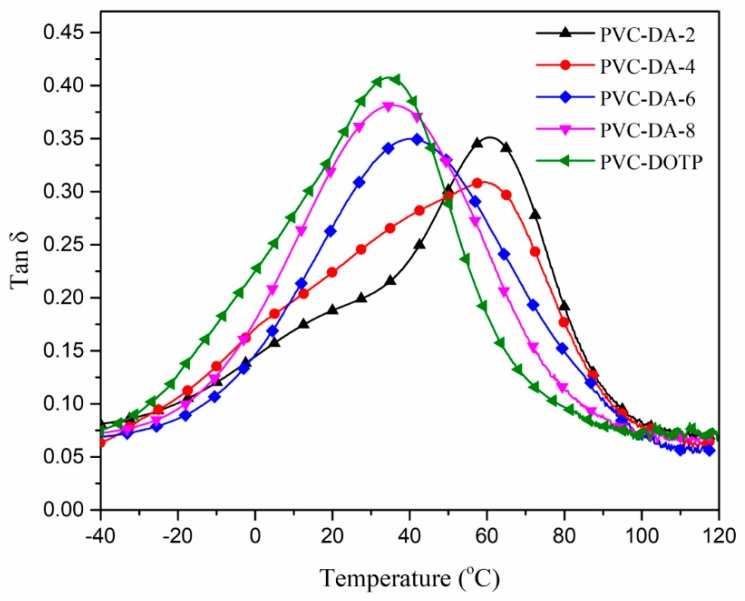
DMA curves of the PVC-DA-2n (2n = 2, 4, 6, and 8) and PVC-DOTP.

It is known that plasticizer can make the material softer and more flexible by reducing the *T*_g_ of the polymer. That means *T*_g_ can be regarded as an index for the plasticizing efficiency of plasticizer. Figure 4 shows the loss tangents (tan δ) of PVC-DA-2n and PVC-DOTP as a function of temperature. It was found that there was only one symmetrical peak located on the DMA curve of PVC-DA-6 or PVC-DA-8, which indicates that the plasticizers of DA-6 and DA-8 were both miscible with PVC matrix. With increasing the number of oxyethyl units, the tan δ peaks shifted from 40.2 (PVC-DA-6) to 35.4 °C (PVC-DA-8), implying that DA-8 has a better plasticization than DA-6. These phenomena can be interpreted using lubricity and free-volume theories. First, the polar groups including carboxylic ester, and oxyethyl units of dimer acid esters strongly interact with the polar fraction of the PVC, which can reduce the friction of the PVC matrix and increase their compatibility. Evidently, DA-8, having more oxyethyl units, exhibited better compatibility than DA-6. Furthermore, the linear oxyethyl units and non-polar alkyl chain on DA-2n can increase the free volume between polymer molecules, resulting in the decline of *T*_g_. Jia et al. [29] also observed that the incorporation of the ether bands into the structure of a castor oil-based plasticizer can efficiently reduce the *T*_g_ of the plasticized PVC.

**Figure 5:**
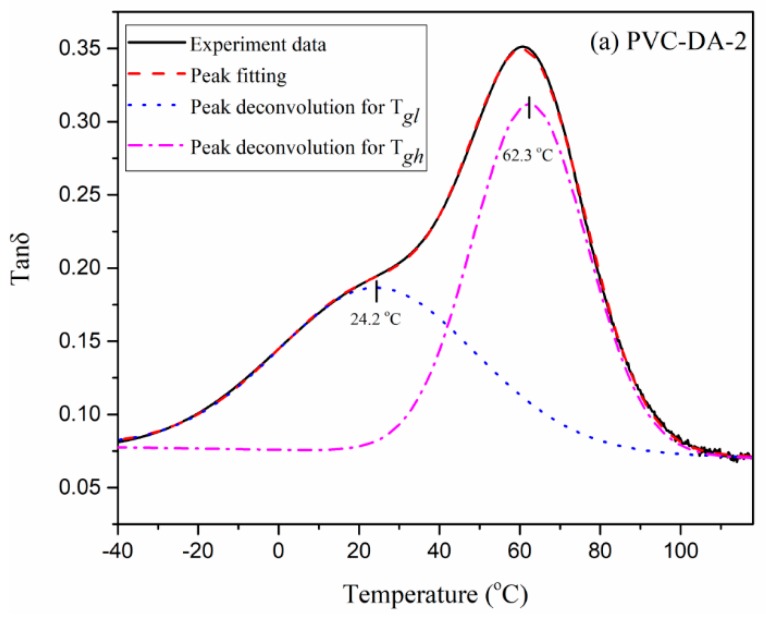

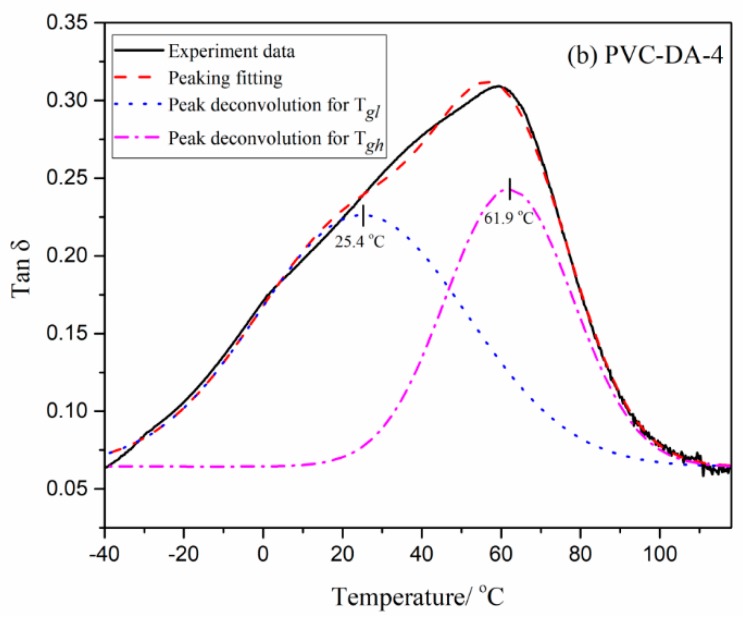
(**a**) Curve deconvolution results of PVC-DA-2. (**b**) Curve deconvolution results of PVC-DA-4.

In addition, the broad and asymmetric tan δ peaks in PVC-DA-2 and PVC-DA-4 can be attributed to phase separation [19]. Deconvolution of the peaks in the tan δ curves of PVC-DA-2 and PVC-DA-4 was performed using the PeakFit software to yield two Gaussian functions for each sample (Figure 5). This result indicates that samples of PVC-DA-2 and PVC-DA-4 have two glass transitions each. One corresponds to the plasticizer-rich region with a low glass transition temperature (labeled as *T*_gl_), and the other corresponds to the PVC-rich region with a high glass transition temperature (labeled as *T*_gh_) [30]. The degree of micro-phase separation can be assessed from the difference of *T*_g_’s (Δ*T*_g_ (*T*_gh_ − *T*_gl_)) [31]. As shown in Table 1, the Δ*T*_g_ value of PVC-DA-4 was smaller than that of PVC-DA-2, indicating that DA-4 was more compatible with PVC than DA-2. This result can be explained by the fact that the interaction between PVC and DA-4 with four oxyethyl units is stronger than that between PVC and DA-2. This gives DA-4 better compatibility. It should be noted that PVC-DA-8 exhibited an even lower *T*_g_ that was almost the same as that of PVC-DOTP (35.4 °C vs. 34.7 °C for peak temperatures of tan δ), indicating that DA-8 could be a substitute for DOTP in PVC plasticization.

### 3.4. Optical Properties of PVC Containing DOTP and DA-2n

**Figure 6:**
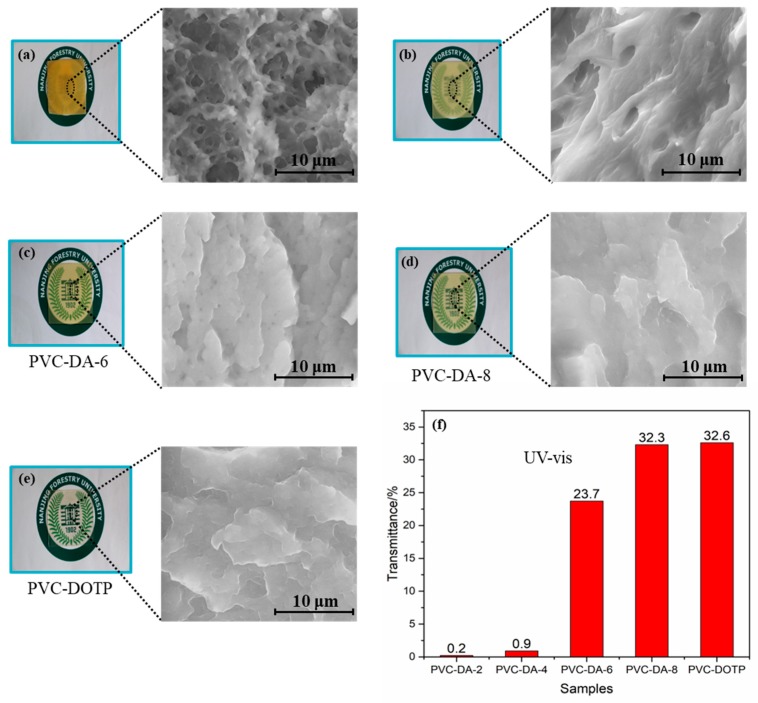
Photographs, SEM images (**a**–**e**), and the UV-vis transmittance (**f**, at a wavelength of 500 nm) of PVC-DA-2n (2n = 2, 4, 6, 8) and PVC-DOTP.

Figure 6 shows the photographs, SEM images, and UV-vis transmittance of PVC-DA-2n and PVC-DOTP. From photographs, it can be found that PVC-DA-6, PVC-DA-8, and PVC-DOTP are relatively transparent. This indicates that these plasticizers have good miscibility with PVC, while the samples of PVC-DA-2 and PVC-DA-4 are optically opaque due to their poor compatibilities. The transparency of the plasticized PVC samples increased from PVC-DA-2 to PVC-DA-8, which was consistent with the UV analysis results (Figure 6f). The SEM micrographs of the cryo-fractured surfaces of PVC-DA-2 (Figure 6a) and PVC-DA-4 (Figure 6b) exhibited many holes, implying that a large-scale micro-sized phase separation exists in these samples. As the number of oxyethyl units in dimer acid esters increased to 6 or 8, the fracture surface of the resulting plasticized PVC samples exhibited fewer holes and was similar to that of PVC-DOTP (Figure 6e). The increased homogeneity of the phase structure with the increase in the number of oxyethyl units was likely attributed to the increasing plasticization effect [32].

### 3.5. Tensile Properties

**Figure 7:**
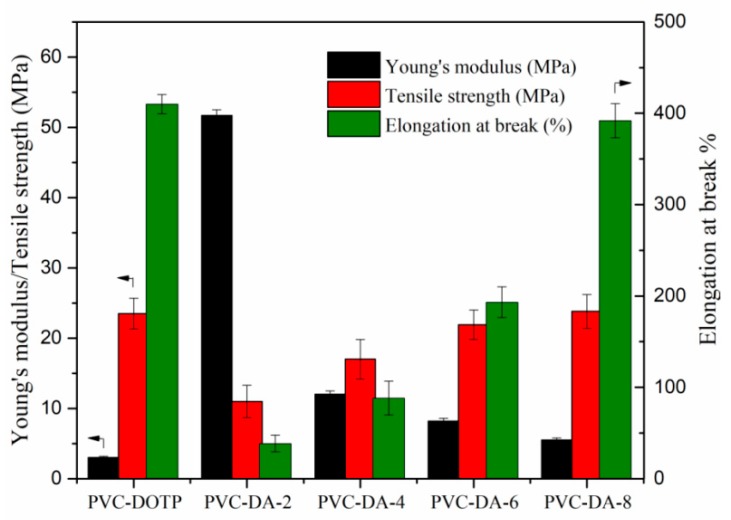
Tensile properties of PVC-DA-2n (2n = 2, 4, 6, 8) and PVC-DOTP.

As shown in Figure 7, PVC-DA-2 exhibited the highest elastic modulus but an elongation of only 38.4%. This result was probably due to the poor compatibility of DA-2, as seen in the aforementioned phase separation. A similar result was also reported by Lee et al. [19]. The strain at the break of the plasticized PVC increased with the number of oxyethyl units in the plasticizer, indicating that these plasticizers were more flexible than DA-2. Compared with PVC-DOTP, PVC-DA-8 exhibited a similar elongation at break and tensile strength but had a higher elastic modulus. The tensile result suggests that PVC-DA-8 has potential as a primary plasticizer to replace DOTP in PVC processing. The satisfactory performance of DA-8 can be attributed to the fact that the polarity of the plasticizer DA-2n increased with the number of flexible oxyethyl units, which consequently improves the compatibility between the plasticizer and PVC. The flexible oxyethyl unit also contributes to the flexibility of the plasticized material.

### 3.6. Thermogravimetric Properties

**Figure 8:**
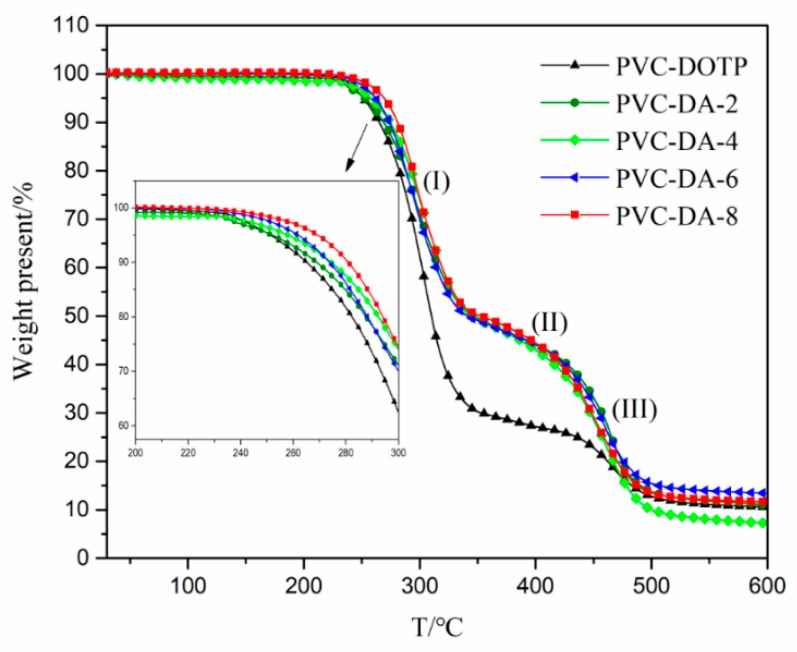
TGA curves of PVC-DA-2n (2n = 2, 4, 6, and 8) and PVC-DOTP.

Figure 8 shows the TGA curves of the PVC-DA-2n and PVC-DOTP samples, and these results are summarized in Table 1. In Figure 8, all the samples displayed a three-stage thermal degradation process under a nitrogen atmosphere. The first stage of degradation (I) between 230 and 340 °C was attributed to the dechlorination of PVC [33]. The materials experienced the largest weight loss in this stage due to the release of HCl and other volatile substances. The second stage of degradation (II) occurred between 340 and 420 °C, where the weight loss was relatively small and the aromatic compounds were formed by the in-sequence cyclization of conjugated polyene [28]. When the temperature was increased to 420 °C, the complex structure decreased and olefins were released (stage (III)) [34]. The degradation temperature corresponding to a 5% weight loss of PVC-DA-2n increased as the number of oxyethyl units increased. This could probably be attributed to good compatibility and increasing of molecular weight. As expected, all PVC-DA-2n samples exhibited a higher *T*_5%_ than PVC-DOTP (Table 1). This result suggests that DA-2n can probably replace DOTP for application in high-temperature environments.

### 3.7. Volatility Test

**Figure 9:**
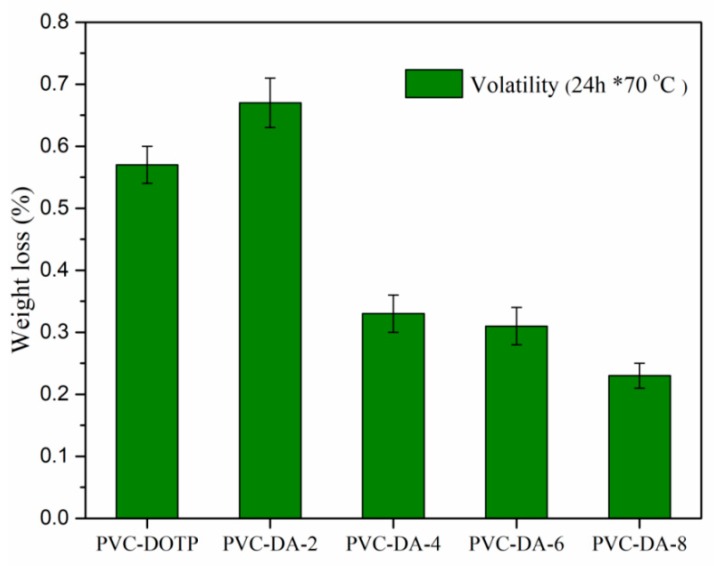
Weight losses of PVC-DA-2n (2n = 2, 4, 6, and 8) and PVC-DOTP.

Figure 9 shows the weight losses of the PVC-DA-2n and PVC-DOTP samples during the volatilization experiment. Except for PVC-DA-2, all the other PVC-DA-2n samples exhibited a lower weight loss than PVC-DOTP during the test. The weight loss tended to decrease as the number of oxyethyl units in the plasticizer increased. The better volatility resistance of the PVC plasticized by DA-2n over DOTP was likely due to their higher molecular weights. However, because of the poor compatibility between DA-2 and PVC, PVC-DA-2 yielded a much higher volatility value than PVC-DOTP. Overall, the PVC plasticized by dimer acid-derived polyethylene glycol methyl ether esters seems more suitable for applications in high-temperature environments.

### 3.8. Leaching Test and Biodegradation

**Figure 10:**
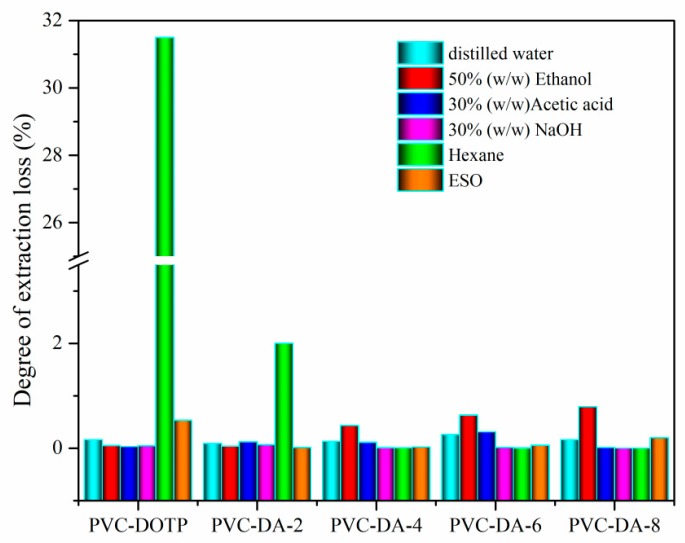
Degrees of extraction loss of PVC-DA-2n (2n = 2, 4, 6, and 8) and PVC-DOTP at 25 °C for 24 h.

Figure 10 shows the results of the leaching tests of the plasticized PVC samples. It was found that there was no significant weight loss of PVC-DA-2n and PVC-DOTP after they were immersed in distilled water, NaOH, acetic acid, and epoxidized soybean oil (ESO). DOTP and DA-2n are insoluble in a water solution or ESO, and they are chemically stable even in the corrosive solution of acetic acid or NaOH under testing conditions (23 ± 2 °C). On the other hand, even though there is the poor compatibility between PVC and DA-4 or DA-2, the high molecular weight of DA-2 and DA-4 can also inhibit their migration from the PVC matrix. In addition, the number of oxyethyl units also affected the migration resistance of PVC-DA-2n. In general, the solvent resistance of PVC-DA-2n increased as the number of oxyethyl units increased because the molecular weight of DA-2n and its compatibility with PVC both increased. Interestingly, when PVC-DA-2n was immersed in 50% (*w*/*w*) ethanol, it showed an opposite trend. This might be attributed to the strong interaction of the hydrogen bond between the solution and oxyethyl unit. It should be noted that all PVC-DA-2n samples exhibited much better resistance than PVC-DOTP in hexane. The combination of the volatility and migration results suggests that the plasticizer DA-8 may outperform DOTP for applications in high temperatures and complex environments. The advantages of DA-2n can increase their application in agricultural film, PVC flooring, or pipes, especially when there may be contact with non-polar solvents.

In addition, the biodegradation property of dimer acid-based plasticizer was studied by comparing the degradation degree of DA-8 and DOTP. The results indicate that 76.5 wt % DA-8 was converted into a micromolecule such as octadecane (mass spectrum was shown in Figure S4a) and stearic acid (mass spectrum shown in Figure S4b), whereas the degradation rate of DOTP was only 5.4 wt %. This result indicates that DA-8 has a better biodegradation property than petro-based DOTP. Chandra et al. [35] also proved that dimer acid polyethylene glycol ester-based derivative has good biodegradability.

## 4. Conclusions

We report a simple, feasible, and efficient method for preparing vegetable oil-based primary plasticizers for PVC. The effects of the number of oxyethyl units on the properties of dimer acid esters-based plasticizers were investigated in detail. It was found that oxyethyl units can efficiently improve the compatibility, plasticization, and thermal stability of plasticized PVC. The polar oxyethyl unit was found to encourage the compatibility between PVC and dimer acid esters-based plasticizers. In addition, increasing plasticization can also be attributed to the good flexibility of oxyethyl units in the vegetable oil-based plasticizer. When the number of oxyethyl units was increased up to eight, the resulting plasticized PVC-DA-8 exhibited similar transparency, tensile properties, and *T*_g_ to PVC-DOTP, as well as higher thermal stability and major migration resistance properties compared to PVC-DOTP. That means that DA-8 with unique oxyethyl units has great potential to act as an environmentally friendly plasticizer that can replace commercial DOTP.

## Figures and Tables

**Table 1:** Thermal and mechanical properties of PVC-DA-2n and PVC-DOTP.

Samples	*T*_5%_^[a]^	*T*_g_^[b]^	Δ*T*_g_ (*T*_gh_ − *T*_gl_) ^[c]^
PVC-DA-2	252.1	-	38.1
PVC-DA-4	258.1	-	36.5
PVC-DA-6	262.5	40.2	0
PVC-DA-8	269.3	35.4	0
PVC-DOTP	251.3	34.7	0

^[a]^ 5% weight loss temperature was detected by TGA; ^[b]^ Glass transition temperature determined by DMA; ^[c]^ calculated based on the results of curve deconvolution from DMA.

## Supplementary Materials

The following are available online at https://www.mdpi.com/2073-4360/11/5/779/s1. Scheme SI. (a) Synthesis of palm oil-based plasticizer [13]; (b) Synthesis of oleic acid-based plasticizer containing polar sulfonyl and carboxylate groups [14]; Scheme SII. Schematic synthetic route of dimer acid. Figure S1. ^1^H NMR spectra of dimer acid (DA) and its’ esters at 6.7–7.10 ppm. Figure S2. ^13^C NMR spectra of DA and its esters. Figure S3. Magnification of the FTIR of PVC samples plasticized by DA-2n. Figure S4. (a) Mass spectrum of octadecane; (b) Mass spectrum of stearic acid.
