# Supplementary material for: Role of the Oxethyl Unit in the Structure of Vegetable Oil-Based Plasticizer for PVC: An Efficient Strategy to Enhance Compatibility and Plasticization
Source: Polymers (Basel). 2019 May 1;11(5):779. doi: 10.3390/polym11050779 (PMC6572382; doi:10.3390/polym11050779)
Supplement: Supplementary file 1 [file polymers-11-00779-s001.zip › polymers-456833-Supplementary/supplementary.docx]

Supporting information

Role of the Oxethyl Unit in the Structure of Vegetable Oil-Based Plasticizer for PVC: An Efficient Strategy to Enhance Compatibility and Plasticization

Jihuai Tan, Bowen Liu, Qinghe Fu, Liwei Wang, Junna Xin and Xinbao Zhu

S1. The procedures for the synthesis of palm oil-based and oleic acid-based plasticizers

**Scheme SI.** (**a**) Synthesis of palm oil-based plasticizer [13]; (**b**) Synthesis of oleic acid-based plasticizer containing polar sulfonyl and carboxylate groups [14].

S2. Synthesis of dimer acid (DA)

**Scheme SII.** Schematic synthetic route of dimer acid.

S3. Characterization of fatty acid, dimer acid (DA) and dimer acid polyethylene glycol methyl ether esters (DA-2n)

**
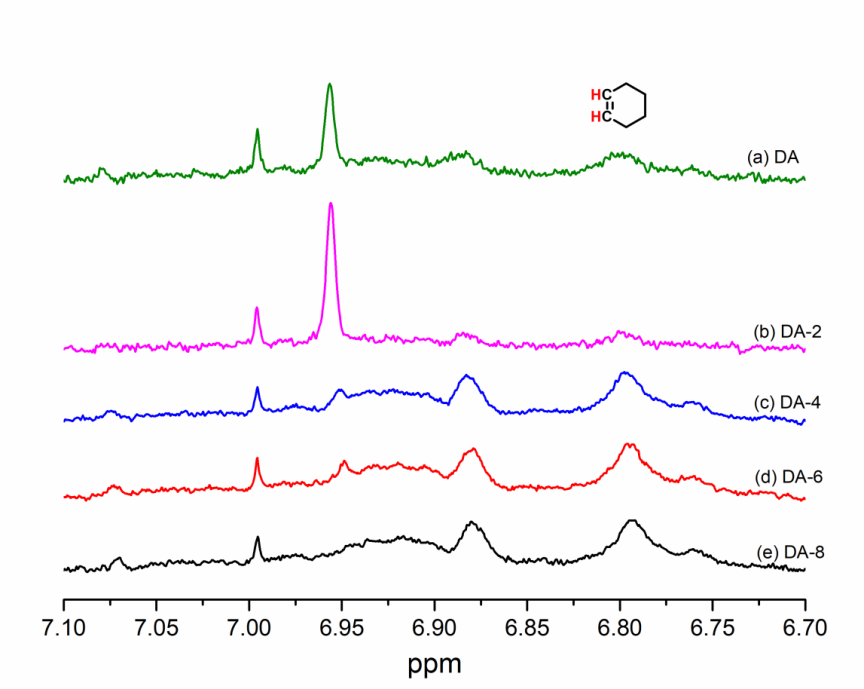
**

**Figure S1.** ^1^H NMR spectra of (**a**) DA, (**b**) DA-2, (**c**) DA-4, (**d**) DA-6 and (**e**) DA-8 at 6.7-7.10 ppm.


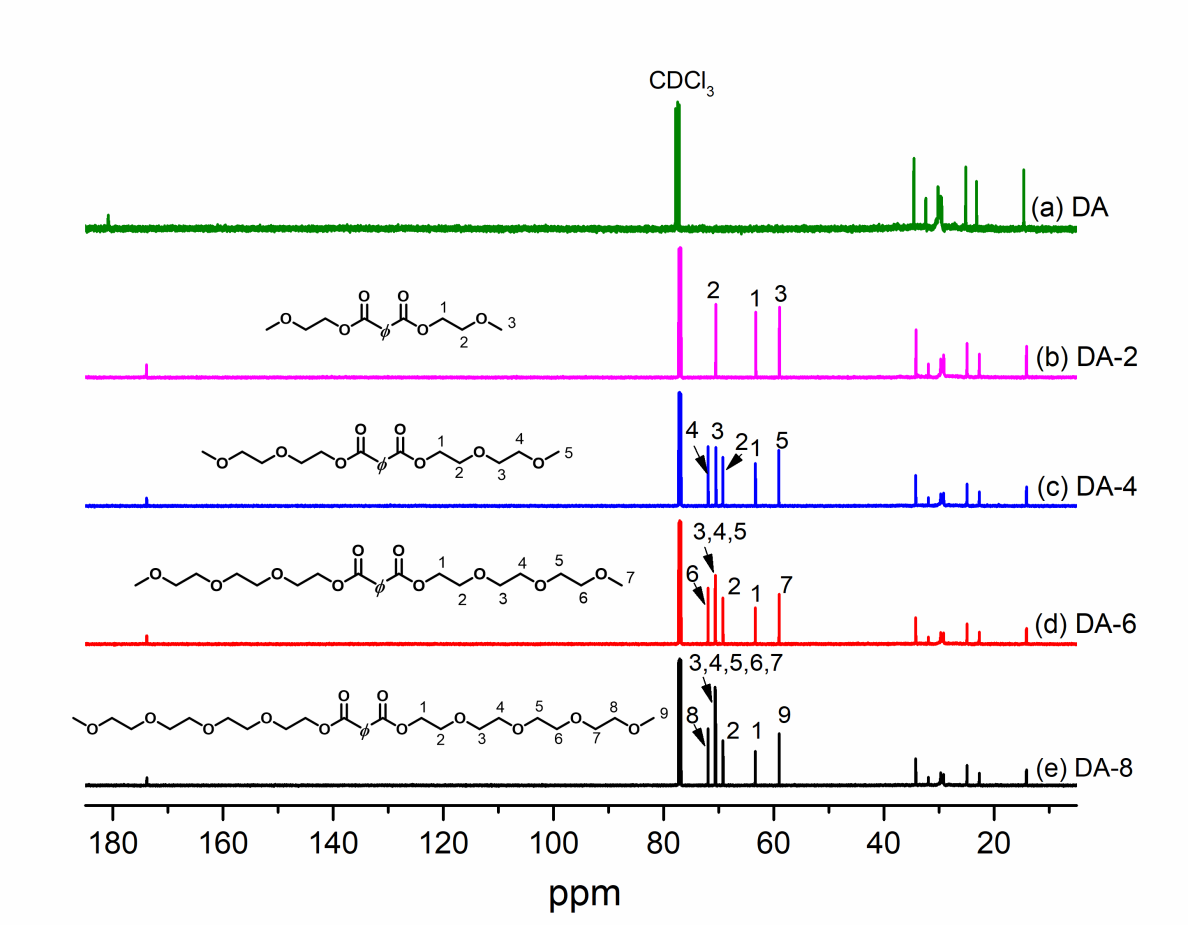


**Figure S2.** ^13^CNMR spectra of (**a**) DA, (**b**) DA-2, (**c**) DA-4, (**d**) DA-6 and (**e**) DA-8.

S4 Magnification on FTIR of PVC samples plasticized by DA-2n

**
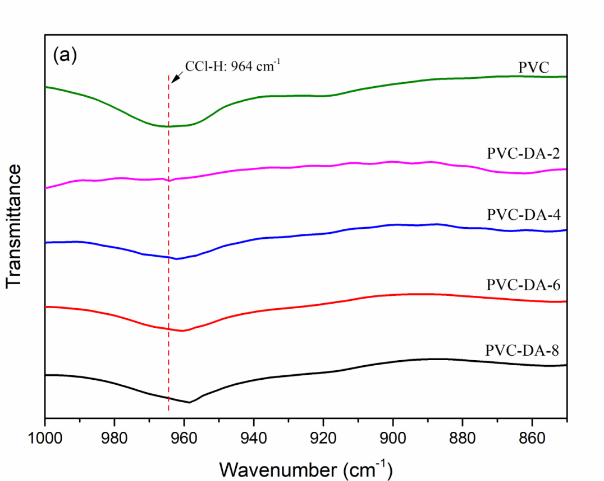

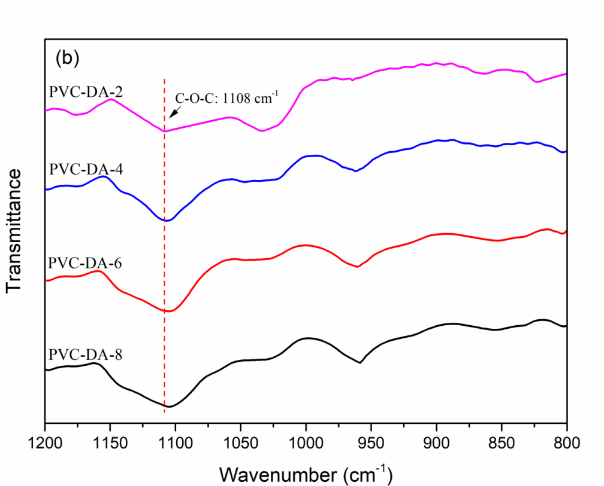
**

**Figure S3.** Magnification on FTIR of PVC samples plasticized by DA-2n.

S5 Mass spectra of degradation product


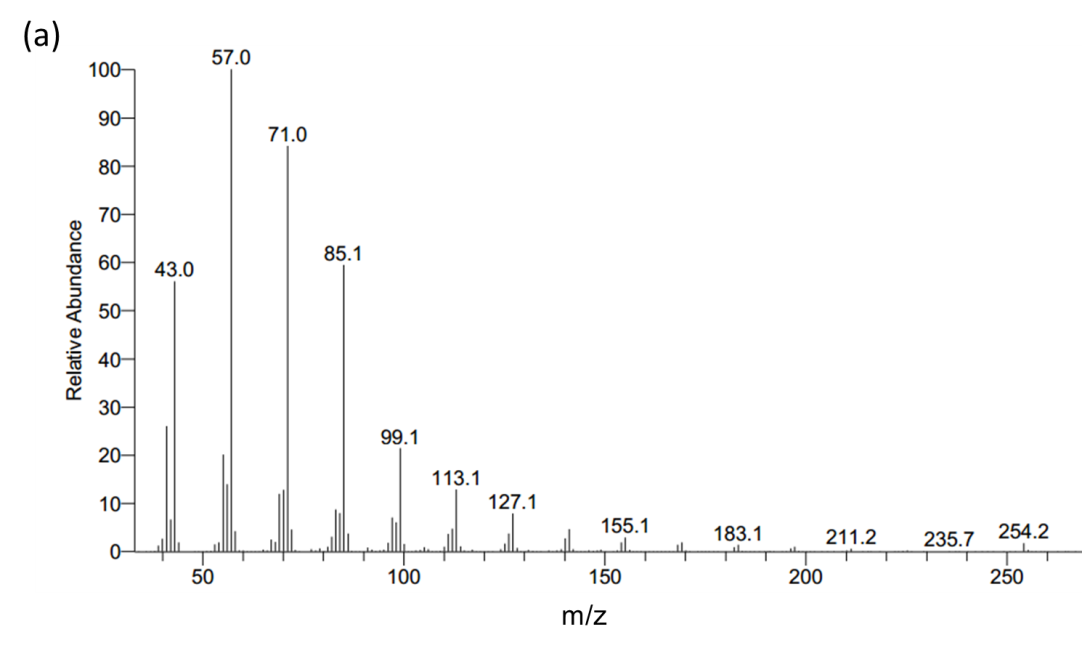


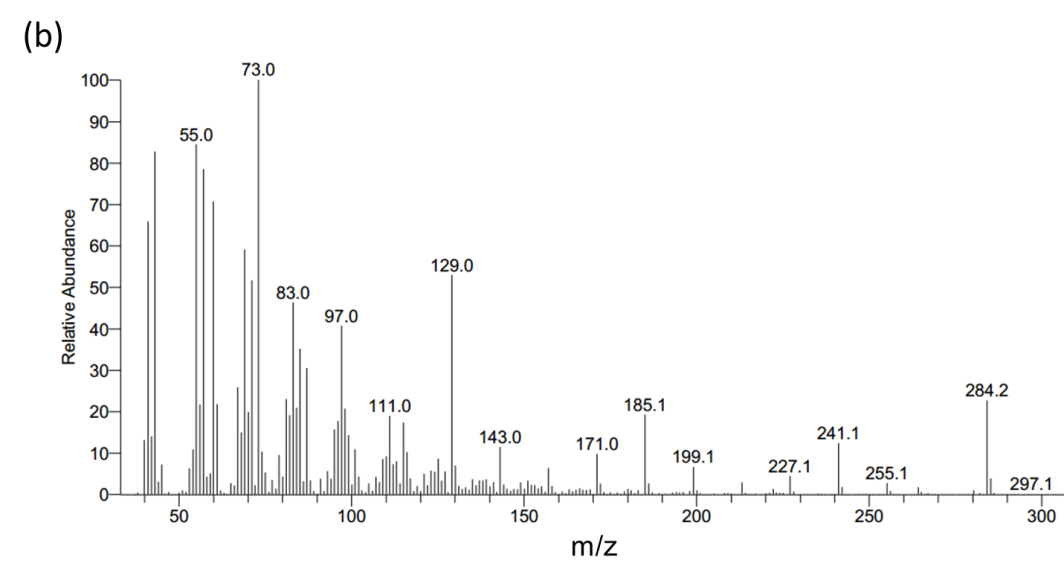


**Figure S4.** (**a**) Mass spectrum of octadecane; (**b**) Mass spectrum of stearic acid.
