# Supplementary figures and images for: Role of the Oxethyl Unit in the Structure of Vegetable Oil-Based Plasticizer for PVC: An Efficient Strategy to Enhance Compatibility and Plasticization
Source: Polymers (Basel). 2019 May 1;11(5):779. doi: 10.3390/polym11050779 (PMC6572382; doi:10.3390/polym11050779)

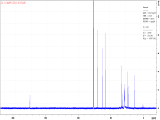

Supplement: Supplementary file 1 [file polymers-11-00779-s001.zip › polymers-456833-Supplementary/NMR/CNMR/DA-2/pdata/1/thumb.png]

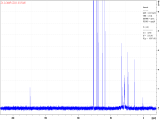

Supplement: Supplementary file 1 [file polymers-11-00779-s001.zip › polymers-456833-Supplementary/NMR/CNMR/DA-4/pdata/1/thumb.png]

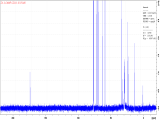

Supplement: Supplementary file 1 [file polymers-11-00779-s001.zip › polymers-456833-Supplementary/NMR/CNMR/DA-6/pdata/1/thumb.png]

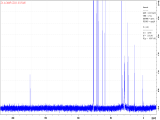

Supplement: Supplementary file 1 [file polymers-11-00779-s001.zip › polymers-456833-Supplementary/NMR/CNMR/DA-8/pdata/1/thumb.png]
